# Effect of Different Levels of Maternally Derived Genotype VII Newcastle Disease Virus-Specific Hemagglutination Inhibition Antibodies on Protection against Virulent Challenge in Chicks

**DOI:** 10.3390/v15091840

**Published:** 2023-08-30

**Authors:** Mei Liu, Xinyue Shen, Yan Yu, Jianmei Li, Jianhua Fan, Xuebo Jia, Yabin Dai

**Affiliations:** Jiangsu Institute of Poultry Sciences, Yangzhou 225125, China

**Keywords:** chick, Newcastle disease, genotype VII-matched vaccine, maternally derived antibody, hemagglutination inhibition antibody, efficacy, survivability, virus shedding

## Abstract

Newcastle disease (ND), caused by the virulent Newcastle disease virus (NDV), is an acute, highly contagious, and economically significant avian disease worldwide. Vaccination is the most effective measure for controlling ND. In recent years, vaccines matched with the prevalent strains of genotype VII have been developed and are now commercially available. These vaccines can provide full protection for chickens against clinical disease and mortality after challenges with genotype VII viruses and significantly decrease virus shedding compared to conventional vaccines belonging to genotypes I and II. Vaccinated hens can transfer antibodies to their offspring through the egg yolk. Maternally derived antibodies can provide passive protection against diseases but can also interfere with vaccination efficacy early in life. This study was conducted on chicks hatched from hens vaccinated with a commercial genotype VII NDV-matched vaccine to investigate the correlation between hemagglutination inhibition (HI) antibody levels in chicks and hens and the decaying pattern of maternally derived HI antibodies, and to evaluate the protective efficacy of different levels of maternally derived HI antibodies against challenge with a virulent NDV strain of genotype VII based on survivability and virus shedding. The HI antibody titers in chicks at hatching were about 1.3 log_2_ lower than those in hens, indicating an antibody transfer rate of approximately 41.52%. The estimated half-life of these antibodies was about 3.2 days. The protective efficacy of maternally derived HI antibodies was positively correlated with the titer. These antibodies could effectively protect chicks against mortality when the titer was 7 log_2_ or higher, but they were unable to prevent virus shedding or infection even at a high titer of 11 log_2_. The obtained results will greatly assist producers in determining the immune status of chicks and formulating appropriate vaccination schedules against ND.

## 1. Introduction

Newcastle disease (ND) is a highly contagious and often severe disease with worldwide distribution that can cause substantial economic losses, and it remains a major threat to the poultry industry around the world. The causative agent of the disease is the virulent Newcastle disease virus (NDV) currently known as *Orthoavulavirus javaense*, which is a negative-sense single-stranded RNA virus and belongs to the genus *Orthoavulavirus*, subfamily *Avulavirinae*, and family *Paramyxoviridae* [1]. Virulent strains are defined by the World Organisation for Animal Health (WOAH) as viruses that have an intracerebral pathogenicity index (ICPI) of 0.7 or higher (2.0 is maximum) or a fusion cleavage site with multiple basic amino acids and phenylalanine at position 117 [2]. The genome of NDV encodes for six structural proteins, nucleocapsid (NP), phosphoprotein (P), matrix (M), fusion (F), hemagglutinin-neuraminidase (HN), the RNA-dependent RNA polymerase (large protein, L), and also for two nonstructural proteins, V and W from P gene editing [3,4]. Among them, HN and F proteins play a major role in virus infectivity and pathogenicity; HN protein is responsible for viral attachment to the host cell, and the F protein is required for viral fusion to the host cell membrane [5]. All NDV isolates characterized to date are antigenically recognized as one single serotype [6]. They are further classified into two classes, class I and class II, based on the complete sequence of the F gene. Class I isolates are lentogenic, possessing only 1 genotype (genotype 1), while class II isolates are composed of 21 genotypes (genotypes I–XXI), and they can be avirulent or virulent [7]. The majority of ND outbreaks worldwide were associated with virulent NDV belonging to genotypes V, VI, VII, and IX of class II.

Since there is no effective treatment for ND, both adequate biosecurity to protect chickens from contracting virulent viruses and proper vaccination to resist virus invasion are required to control the disease [8,9]. NDV strains for conventional commercially available vaccines belong to genotypes I (Ulster, QV4) and II (LaSota, B1, VG/GA). They are more phylogenetically divergent from prevalent strains in the last two decades, among which the genotype VII strain is predominant in China and some other countries in Asia [10,11,12,13,14,15,16]. The vaccines heterologous to prevalent strains lead to incomplete protection, characterized by persistent virus shedding and atypical clinical symptoms in the vaccinated flocks [17,18,19,20]. Decreasing the amount of virus shed from vaccinated birds has been an important consideration in ND control. Previous studies have reported that vaccines homologous to the prevalent strains can induce a higher level of humoral immune response, and are more efficient in reducing the number of birds shedding virus and the amount of virus shed from birds than conventional vaccines, potentially reducing the risk of horizontal transmission of virulent NDV to some extent; despite this, both genotype-matched and conventional vaccines can provide good protection against obvious clinical disease and mortality from field viruses [17,19,20,21].

HN and F proteins are the main targets of the immune response against NDV that provides protection from virulent NDV [22,23]. Vaccine-induced antibodies against the HN are responsible for blocking viral attachment, whereas antibodies against the F glycoprotein can inhibit viral fusion with the host cell membrane. Protection against NDV is highly correlated with the hemagglutination inhibition (HI) levels of serum antibodies commonly estimated by the HI test [2,24,25]. When the level of serum HI antibody titers increases in vaccinated birds, the number of infected birds and the amount of virulent NDV shed would decrease [6,26,27,28].

To effectively control the occurrence and prevalence of ND, the genotype-matched vaccines made of attenuated mutant viruses derived from prevalent virulent genotype VII isolates have been developed by reverse genetics and have already been commercialized in the form of live and inactivated vaccines in some countries [29,30,31]. Compared to the LaSota vaccine, these genotype VII NDV-matched vaccines can induce a faster and stronger antibody response, fully protecting chickens from clinical disease and mortality after the challenge with genotype VII viruses, and significantly decreasing virus shedding [18,32,33]. Breeder hens vaccinated with live attenuated or inactivated NDV vaccine can transfer antibodies to their progeny through the egg yolk. Maternally derived antibodies (MDA) are protective and can protect the chicks against field viruses for the critical first few weeks of their life when their own immune systems are not yet fully matured [34,35,36], avoiding the energetic cost of resisting infection by chicks and allowing chicks to keep this energy for growth and further development of the immune system [37,38,39]. However, besides these benefits, MDA can also interfere with the induction of specific active immune responses to early vaccination with live attenuated vaccine if the chicks are vaccinated in the presence of a high level of MDA [40,41,42,43,44,45,46]. Indeed, MDA can reduce the antigenic presentation via fixation, and by hiding specific epitopes of the vaccine virus, and it can inhibit the replication of the vaccine virus by neutralizing it, which consequently hampers specific active immune responses [34,47]. Consequently, MDA interference is of major concern for the poultry industry regarding this negative impact on the early vaccination against ND.

Under the background that the genotype VII NDV-matched vaccines have gradually been widely used, this study was undertaken to investigate the correlation between HI antibody levels in chicks and vaccinated hens and the decaying pattern of maternally derived HI antibodies and to evaluate the protective efficacy of different levels of maternally derived HI antibodies against a virulent challenge based on survivability and virus shedding. The results obtained in the present study may find direct application in formulating strategies for protecting chicks from ND, especially during the first few weeks of age before the generation of active immune protection.

## 2. Materials and Methods

### 2.1. Virus Strain, Antigen and Antiserum

The filed NDV virulent strain chicken/Jiangsu/JSC0804/2008 (JSC0804) (GenBank accession numbers: MT162178 and MT162179) was obtained from the pathogen inventory in Jiangsu Institute of Poultry Sciences (Yangzhou, China). It was isolated from an outbreak of ND in a flock and identified as a velogenic genotype VII virus with an ICPI of 1.69 [48]. The strain was successively passaged for two generations in 9- to 10-day-old specific pathogen-free (SPF) chicken embryos via allantoic cavity inoculation. The harvested infective allantoic fluid was clarified via centrifugation at 1000× *g* for 10 min. It was titrated to determine 50% embryo lethal doses (ELD_50_) with SPF embryos and subpackaged prior to being stored at −80 °C for future use.

The standard HI antigen and positive antiserum of genotype VII NDV strain were obtained from Qingdao Yebio Bioengineering Co., Ltd. (Qingdao, China).

### 2.2. SPF Chicken Embryo

SPF fertile chicken eggs were purchased from Zhejiang Hengda Agricultural Development Co., Ltd. (Yuyao, China). They were incubated for 9 to 10 days in the incubator and used for virus propagation, virus titration, and virus isolation.

### 2.3. Experimental Birds

All breeder hens and newly hatched chicks used for this study were obtained from a breeder flock of green-shell chickens in a breeder farm with good biosafety practices. The breeder flock had been vaccinated for ND at 1 and 4 weeks of age with the combined live attenuated vaccine against ND and infectious bronchitis (VG/GA strain + H120 strain) (Boehringer Ingelheim (China) Investment Co., Ltd., Shanghai, China) intraocularly and intranasally and at 5 and 15 weeks of age with the inactivated recombinant NDV vaccine (A-VII strain, genotype VII) (Qingdao Yebio Bioengineering Co., Ltd., Qingdao, China) intramuscularly. At 20 weeks of age, oropharyngeal and cloacal swabs were collected from 30 randomly selected chickens in the flock for monitoring the infection of field virus, and no NDV was isolated from swab samples with 10-day-old SPF chicken embryos.

### 2.4. Experimental Design

#### 2.4.1. Experiment 1

To investigate the relationship of HI antibody levels against NDV between breeder hens and their progeny, eight 22-week-old hens with different antibody levels were chosen for the experiment. Each hen was placed in a single egg-laying cage and artificially inseminated twice weekly. After a serum sample from each hen was taken for determination of the HI antibody titers, a collection of the fertile eggs they laid was initiated. The eggs were collected and labeled for ten consecutive days afterwards. They were incubated in 2 batches, every five days per batch. At 18 days of incubation, the eggs laid by the same hens were placed in a nylon net bag for hatching. Sera of the hatched chicks were sampled within 12 h after hatching. The hens were bled via the wing vein, and the chicks via the jugular vein. All serum samples were stored at −20 °C till processing. At the end of the experiment, the sera from hens and chicks were simultaneously detected for antibody titers via HI test.

#### 2.4.2. Experiment 2

The objective of this experiment was to determine the decaying pattern of maternally derived HI antibodies against NDV. Fifty newly hatched chicks from the breeder flock at 22 weeks of age were reared for 35 days maintaining all the hygienic measures in an isolated and environmentally controlled room. Commercial feed and water were supplied ad libitum. The chicks were not vaccinated, and no medication was administered during the experiment period. Beginning at hatching, 20 birds were randomly selected and bled for serological detection at different intervals varying from 2 to 4 days up to 35 days of age. The birds were bled via the jugular vein before 6 days of age and via the wing vein thereafter. Serum samples were stored at −20 °C until analysis. At the end of the experiment, sera samples from chicks were detected simultaneously for HI antibody levels by HI test.

#### 2.4.3. Experiment 3

In this experiment, chicks with different antibody levels were challenged to evaluate the efficacy of maternally derived HI antibodies to NDV against virulent viruses based on survivability and virus shedding after the challenge. Three hundred 1-day-old chicks from the breeder flock at 25 weeks of age were identified by wing bands and reared according to the method mentioned above. In order to obtain the chicks with a range of antibody levels, challenges were performed at 5, 10, 15, and 20 days of age, respectively. Before each challenge, a certain number of apparently healthy birds with similar body weights were randomly selected and bled for detection of antibody levels against NDV by HI test. Immediately after the antibody levels were determined, the birds were grouped according to the HI titers and transferred to isolation facilities. They were challenged with a JSC0804 strain of 0.1 mL (10^6^ EID_50_) per bird via the intraocular and intranasal routes, half administered into the eyes and another half into the nostrils. All birds were inspected a minimum of two times daily for the development of clinical signs of the disease and for mortality until 21 days post-challenge (pc). The dead birds were immediately subjected to detailed postmortem examination, and their tissue samples, such as brain, small intestine, liver, spleen, pancreas, lung, and laryngotrachea, were collected aseptically for virus reisolation.

To assess virus shedding after the challenge, for groups with the HI titers of 4 log_2_ and 5 log_2_, beginning on the first day after the challenge, oropharyngeal and cloacal swabs were taken from each surviving bird every 2 days until 31 days pc for virus isolation, while swabs were taken only at 5 days pc for other groups. Swabs were placed in Eppendorf tubes containing 0.75 mL PBS (0.01 mol/L, pH 7.4). At 15 days pc, all surviving birds were bled for serological testing. The swab and serum samples were stored at −20 °C and the tissue samples at −80 °C until further use.

### 2.5. Virus Isolation

Pooled tissue (brain, small intestine, liver, spleen, pancreas, lung, and laryngotrachea) sample from each dead bird was finely minced and added with PBS (0.01 mol/L, pH 7.4) at a ratio of 1:4 (*v*/*v*). The swab and three times freeze–thawed pooled tissue samples were cleared by centrifugation at 6700× *g* for 5 min. Penicillin and streptomycin were added to the supernatants to final concentrations of 2000 IU/mL and 2 mg/mL, respectively, and inoculated into 9- to 10-day-old SPF embryos via allantoic cavity route, using 2 embryos per sample (0.2 mL/embryo). The inoculated embryos were incubated for 120 h and candled twice daily after 24 h post-inoculation. The allantoic fluids of embryos that died within 24 to 120 h and of those still alive at 120 h were harvested and tested for hemagglutination (HA) activities. Fluids from the dead embryos that resulted in a negative reaction were re-tested. If the third-generation fluid of a sample still tested HA negative, its virus isolation was determined to be negative.

### 2.6. HA and HI Tests

HA activity of allantoic fluid and HI antibody titer against NDV in serum were determined by HA and HI tests, respectively, in V-bottomed 96-well microtiter plates according to the procedure recommended in the Terrestrial Manual of WOAH [2]. The HI test was performed using 4 HA units of the genotype VII NDV antigen.

### 2.7. Statistical Analysis

Statistical analysis was performed with the help of IBM SPSS Statistics for Windows, version 22.0 (IBM Corp., Armonk, NY, USA). Single-factor analysis of variance (ANOVA) test and Pearson’s chi-square test were used for comparisons. *p* < 0.05 was considered statistically significant.

## 3. Results

### 3.1. Correlation between HI Antibody Levels in Chicks and Breeder Hens

The HI antibody levels against NDV were compared between hens and their offspring. The results showed that the HI titers of chicks at hatching were about 2- to 3-folds (1.1 log_2_ to 1.6 log_2_ with a mean of 1.3 log_2_) lower than those of their parent hens. The transfer rates of HI antibodies from hens to their chicks ranged from 35.71% to 47.22%, with a mean of 41.52%. There was no significant difference in the transfer rates of antibodies among hens with different antibody levels (*p* > 0.05) (Table 1).

### 3.2. Decaying Pattern of Maternally Derived HI Antibodies in Chicks

**Figure 1:**
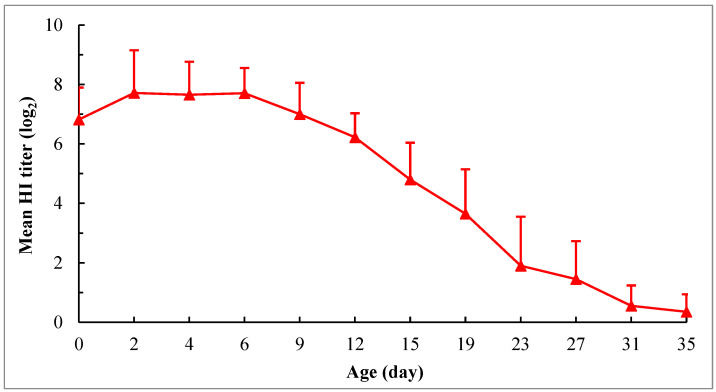
Decaying pattern of maternally derived HI antibodies against NDV in chicks. The chicks were hatched from the hens at 22 weeks of age. The hens were vaccinated at 1 and 4 weeks using the combined live attenuated vaccine against ND and infectious bronchitis (VG/GA strain + H120 strain) (intraocularly and intranasally) and at 5 and 15 weeks using the inactivated recombinant NDV vaccine (A-VII strain, genotype VII) (intramuscularly). The chicks were not vaccinated, and no medication was administered during the experimental period. The HI test was performed using 4 HA units of the genotype VII NDV antigen. For each time point, the HI antibody titers are expressed as means ± SD based on the 20 chicks randomly selected from 50 chicks.

Beginning at hatch, the maternally derived HI antibody levels against NDV in chicks were successively detected at various intervals for 35 days. The mean HI titer of birds at hatching was (6.8 ± 1.08) log_2_. At 2 days of age, the antibody level slightly rose (about 0.9 log_2_ higher than that at hatching) and maintained this level until 6 days of age. Thereafter, the antibody level declined linearly as birds aged, and the antibodies were virtually depleted in all birds at 35 days of age (Figure 1). Since depletion of the HI antibodies was found in about 30% of birds at 23 days of age, the half-life of maternally derived HI antibodies was estimated based on the change of titers between 6 and 19 days of age. The calculated value was about 3.2 days.

### 3.3. Protective Efficacy of Different Levels of Maternally Derived HI Antibodies against the Challenge with Virulent NDV

A total of 288 apparently healthy chicks with different levels of maternally derived HI antibodies were chosen from 300 birds for the challenge in four batches at 5, 10, 15, and 20 days of age, respectively.

#### 3.3.1. Clinical Signs and Gross Lesions

The results showed that the survival rate after the challenge tended to increase with an increase in HI antibody titer on the day of the challenge. During the observation period of 21 days, all birds in groups with HI titers ≥ 9 log_2_ withstood the challenge of infection, while all of those in groups with HI titers ≤ 3 log_2_ died. There was no significant difference in survival rate among groups with HI titers ≥ 7 log_2_ (*p* < 0.05) though deaths did occur in groups with HI titers of 7 log_2_ and 8 log_2_. Otherwise, at the same HI titer, no significant difference was found in survival rate among birds of different ages (*p* > 0.05) (Table 2).

Generally, the birds began to show clinical symptoms such as depression, dyspnea, anorexia, polydipsia, and watery, greenish-white diarrhea 3 or 4 days pc, and some of them died or developed varying degrees of neurological signs such as head or muscular tremors, torticollis, and paralysis of one wing or one leg afterward. Neurological signs usually became obvious at approximately 12 days pc, and maintained in about 30.0% (3/10), 23.5% (4/17), 2.2% (1/45), and 2.3% (1/44) of the surviving birds in groups with HI titers of 4 log_2_, 5 log_2_, 7 log_2_, and 9 log_2_, respectively, at the end of the experiment (Table 2). In groups with HI titers of 4 log_2_ and 5 log_2_ for investigation of the persistence of virus shedding, all birds with neurological signs that survived at 21 days pc remained alive up to 31 days pc, although they grew poorly and their symptoms were not alleviated.

**Figure 2:**
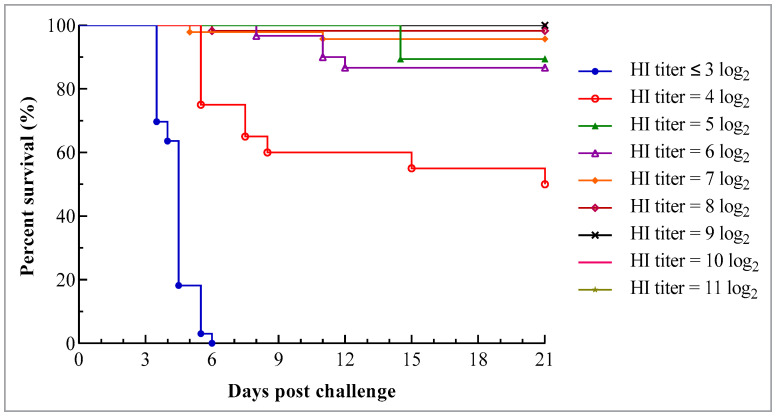
Survival curve of chicks with different levels of maternally derived HI antibodies following challenge with virulent genotype VII NDV strain JSC0804. The chicks were hatched from the hens at 25 weeks of age. The hens were vaccinated at 1 and 4 weeks using the combined live attenuated vaccine against ND and infectious bronchitis (VG/GA strain + H120 strain) (intraocularly and intranasally) and at 5 and 15 weeks using the inactivated recombinant NDV vaccine (A-VII strain, genotype VII) (intramuscularly). Each chick was challenged with 10^6^ ELD_50_ JSC0804 strain intraocularly and intranasally and the survival status was monitored twice daily for 21 days.

In groups where the death occurred, the death time varied with the antibody level on the day of the challenge, and it was slightly delayed with an increase in antibody level. All birds in the group with the HI titer ≤ 3 log_2_ died 4 to 6 days pc, while initial deaths in other groups occurred 5 or more days later (Figure 2).

At necropsy, except for a few birds with the HI titer ≤ 3 log_2_ in which no obvious gross lesion was observed, most dead birds showed specific pathological lesions of ND, such as congestion or hemorrhages on the meninx and in the brain, congestion or hemorrhages in the caudal pharynx and tracheal mucosa, a slightly enlarged, hemorrhagic or mottled spleen, hemorrhagic and necrotic pancreas, extensive hemorrhages in the mucosa of the small intestine, and hemorrhages at the tip of the glands or petechiae in the mucosa of the proventriculus. With 10-day-old SPF embryos, the viruses could be recovered from the tissue samples of dead birds, and their HA activity could be inhibited by NDV antiserum.

#### 3.3.2. Virus Shedding

**Figure 3:**
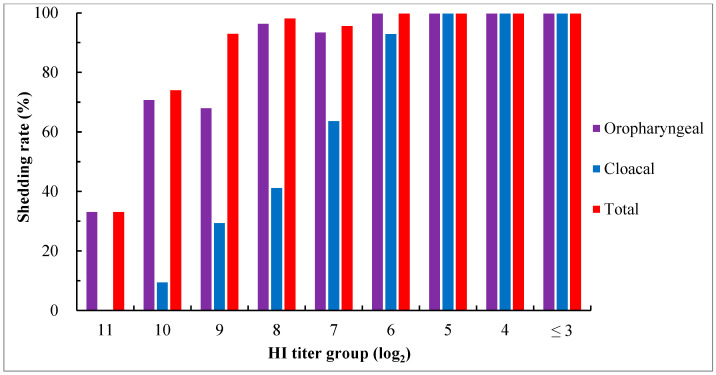
Virus shedding in chicks with different levels of maternally derived HI antibodies at day 5 after the challenge with virulent genotype VII NDV strain JSC0804. Viruses in swabs were detected via virus isolation using 9- to 10-day-old SPF embryos. Oropharyngeal shedding rate = the number of birds positive in virus isolation from oropharyngeal swabs/the number of surviving birds × 100%; cloacal shedding rate = the number of birds positive in virus isolation from cloacal swabs/the number of surviving birds × 100%; total shedding rate = the number of birds positive in virus isolation from oropharyngeal or cloacal or both swabs/the number of surviving birds × 100%.

For determining the virus shedding in groups with different maternally derived HI antibody levels, oropharyngeal and cloacal swabs were taken from all surviving birds at 5 days pc for virus isolation using 10-day-old SPF embryos. The results showed that virus shedding was detected in all groups, even in the group with an HI titer as high as 11 log2. The oropharyngeal, cloacal, and total shedding rates increased with a decrease in maternal HI antibody levels. In groups with HI titers ≤ 5 log_2_, virus shedding was detected from both oropharyngeal and cloacal swabs in all birds, while it was more frequently found in oropharyngeal than in cloacal swabs in other groups (Figure 3).

**Figure 4:**
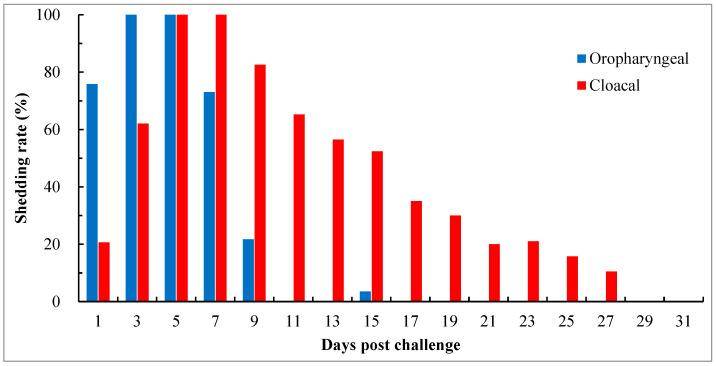
Pattern and duration of virus shedding in chicks with maternally derived HI antibody titers of 4 log_2_ and 5 log_2_ after challenge with virulent genotype VII NDV strain JSC0804. Viruses in swabs were detected by virus isolation using 9- to 10-day-old SPF embryos. Oropharyngeal shedding rate = the number of birds positive in virus isolation from oropharyngeal swabs/the number of surviving birds × 100%; cloacal shedding rate = the number of birds positive in virus isolation from cloacal swabs/the number of surviving birds × 100%. The shedding status was monitored for 31 days.

To investigate the pattern and duration of virus shedding, thirty-nine birds in groups with the HI titers of 4 log_2_ and 5 log_2_ were consecutively detected for 31 days pc. Oropharyngeal shedding began on day 1 pc, peaked on days 3 to 5, and was cleared in all birds by day 11, except for occasional detection on day 15 in one of 27 birds with an HI titer of 4 log_2_. Cloacal shedding could persist for an extended period. The onset of the condition occurred on day 1 pc, peaked on days 5 to 7, and was cleared in all birds by day 29 (Figure 4). Among the birds that survived throughout the experiment, the shedding duration varied among individual birds, ranging between 9 and 25 days, with an average of around 15 days. Some of the birds exhibited discontinuous or intermittent shedding.

#### 3.3.3. Serological Response

**Figure 5:**
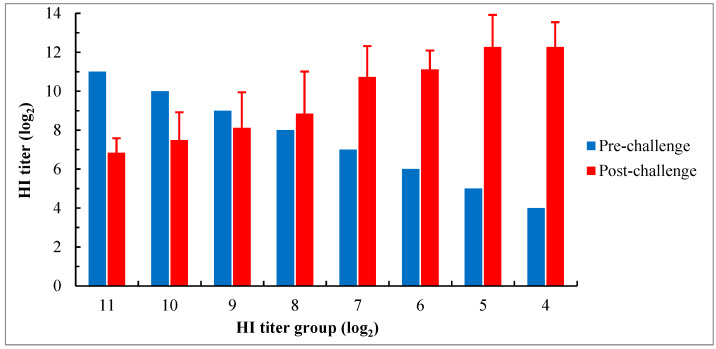
Serological response of chicks with different levels of maternally derived HI antibodies 15 days after the challenge with genotype VII NDV strain JSC0804. All birds in the group with the HI titer ≤ 3 log_2_ died before 6 days pc. The HI test was performed using 4 HA units of the genotype VII NDV antigen. The HI titers after the challenge are shown as means ± SD.

Except for the group with the HI titer ≤ 3 log_2_ in which all birds died 6 days pc, serum samples were collected from all surviving birds in other groups at 15 days pc for examination of serological responses using the HI test with the genotype VII NDV antigen. The results showed that the mean HI antibody titers in groups with titers ranging from 4 log_2_ to 8 log_2_ increased to varying degrees after the challenge compared to their titers before the challenge, and the magnitude of increase was gradually decreased with an increase in titer before the challenge. However, in groups with titers ≥ 9 log_2_, the mean titers after the challenge were lower than their titers before the challenge, even though the virus shedding was also detected in these groups. Moreover, in groups with titers ranging from 8 log_2_ to 10 log_2_, the coefficient of variance (CV) values of HI titer after the challenge were more than 15% (19.2% to 24.5%), which were higher than the values observed in other groups. It was the highest in the group with a titer of 8 log_2_, reaching 24.5% (Figure 5).

## 4. Discussion

Vaccination for protecting chickens from ND is routinely practiced throughout the world. As ND is endemic in China, most breeder farms implement multiple vaccinations with live and inactivated vaccines, even during the production stage. Although there is no universal vaccination schedule for breeders, it is generally accepted to prime the breeders with live attenuated vaccine, followed by boosters with inactivated vaccine to achieve high antibody levels. It was well characterized by several researchers that the vaccinated breeder hens can deposit their circulating antigen-specific antibodies into the yolk before egg laying and hence transfer to the embryo’s and then to the chick’s circulation via receptors on the yolk sac membrane [49,50,51,52]. In this study, the breeder hens from which the chicks were hatched had received two doses of the live attenuated genotype II vaccine (VG/GA strain) early in their life followed by two doses of the inactivated recombinant genotype VII NDV vaccine (A-VII strain). Inactivated vaccines generally induce higher, longer-lasting humoral immunity than live attenuated vaccines. When the experiments were performed, the breeder hens were over 22 weeks of age, and therefore, their circulating antibodies induced by the vaccine, as well as those passed down to their offspring, were believed to be primarily specific to genotype VII NDV.

It is well known that MDA can provide early protection from diseases but also interfere with vaccination efficacy in chicks. Therefore, understanding the level of MDA in newly hatched chicks and its decay pattern is essential for assessing the susceptibility of the flock to infection and predicting the timing of vaccine application without interference with the response of birds to active immunization. MDA levels in chicks are positively correlated with the condition of their mothers. In the present study, the HI antibody titers of chicks at hatching were about 1.3 log_2_ lower than those of the corresponding breeder hens. The transfer rate of HI antibodies from hens to their chicks ranged from 35.71% to 47.22% with a mean of 41.52%, and there was no significant difference observed among hens with different HI antibody titers (*p* > 0.05). In a previous study, Gharaibeh et al. [53] reported NDV antibodies had a transfer rate of 29.2% in a broiler breed based on the antibody levels of 1-day-old chicks. Meanwhile, based on the antibody levels of chicks three days post-hatching when the MDA level typically peaked after hatching, Hamal et al. [54] found the transfer rates of NDV antibodies (IgY isotype) were 31.3% and 36.0% for two different lines of broiler breeds, respectively, with no difference between the lines. Adeleke et al. [55] demonstrated that three local breeds in Nigeria had higher transfer rates, ranging from 88.7% to 96.4%, compared to 34.7% in an exotic broiler breed. The variation in the transfer rate observed across studies may be due to the difference in breeds, the age of chicks, or the serological methods that were used. In this study, the transfer rate was approximately 75% when calculated based on the peak titer of maternally derived HI antibodies in 2-day-old chicks, which was about 0.9 log_2_ higher than the titer in chicks at hatching. Furthermore, the half-life of maternally derived HI antibodies was about 3.2 days in the present study. This finding is partially in agreement with the observations of Wang et al. [56], who reported that half-lives of those antibodies were 2.19 days in layer chicks and 3.10 days in broiler chicks. However, Rahman et al. [36], Jalil et al. [57], and Gharaibeh and Mahmoud [58] estimated that half-lives of maternally derived HI antibodies in broiler chicks were about 5, 7, and 6.3 days, respectively. Nevertheless, the results obtained in this study will be helpful for producers to extrapolate the antibody levels in chicks at hatching and during the next several weeks based on the antibody status in parent flocks.

In this study, challenge experiments were conducted on chicks aged from 5 to 20 days old. However, the susceptibility to infection was essentially consistent among birds, as no age difference was found in survivability after the challenge within groups where birds of different ages with the same maternally derived HI antibody titer were included. The obtained results demonstrated a positive correlation between the presence of antibody titers at challenge and protection from disease. During the 21-day observation period, all birds in groups with HI titers ranging from 9 log_2_ to 11 log_2_ survived, while none of the birds in groups with HI titer ≤ 3 log_2_ were protected after the virulent challenge. Although not all birds in the groups with the HI titers of 7 log_2_ and 8 log_2_ withstood the challenge, their survival rates were not significantly different from those in groups with titers ranging from 9 log_2_ to 11 log_2_ (*p* < 0.05). This suggests that a maternally derived HI antibody titer of 7 log_2_ or higher could provide effective protection against the virulent challenge. The result obtained in this study is consistent with that reported by Jalil et al. [57], despite the use of different vaccines and challenge strains. It should be noted that this experiment was conducted under ideal conditions. In the field, birds may encounter various exacerbating conditions, such as poor feeding management, multipathogen infections, and immunosuppressive factors, which may lead to greater mortality in birds following infection with virulent NDV. Noticeably, some of the surviving birds maintaining neurological signs at the end of this experiment may die from the difficulty of accessing food and water under field conditions.

NDV is mainly spread through horizontal transmission. Infected birds shed viruses in their oropharyngeal secretions and fecal matter. Susceptible birds may become infected by ingesting contaminated feed and water sources, or by inhaling contaminated dust or aerosolized virus. In addition to the standard observation of morbidity and mortality after the challenge, the reduction in virus shedding has been an important indicator for evaluating the efficacy of vaccines. In the present study, the effect of maternally derived HI antibodies against virus shedding was evaluated. The results showed that at 5 days pc, the number of chicks shedding virus tended to decrease with an increase in antibody levels. However, maternally derived HI antibodies were unable to protect birds from virus shedding, even when the titer was as high as 11 log_2_. Among birds possessing antibody titers of 4 log_2_ and 5 log_2_, the challenge viruses were initially shed primarily in oropharyngeal secretions, peaking 3 to 5 days pc, with cloacal shedding occurring later. The duration of oropharyngeal shedding was approximately 9 days, while cloacal shedding could persist until 27 days pc, even though high levels of humoral HI antibodies ranging from 12 log_2_ to 15 log_2_ were yielded in birds 15 days after the challenge. The shorter duration of oropharyngeal shedding may be attributed to the inhibition of virus replication from rapid and strong local mucosal immune responses induced at the sites of challenge. Some previous studies demonstrated that genotype VIId strains can induce more severe lesions in the spleen and thymus when compared with virulent strains of other genotypes (genotypes I, IV, V, and IX), and such lesions are associated with a significantly higher level of virus replication in the tissues [59,60,61,62]. Therefore, a large quantity of viruses in tissues may take more time to release into the intestine and require a greater number of antibodies to inhibit infection or replication. This may be the reason for the prolonged cloacal shedding.

Compared to the antibody titers of chicks on the day of the challenge, the mean HI titers in groups with titers ranging from 4 log_2_ to 8 log_2_ increased 15 days after the challenge. However, in groups with titers ≥ 9 log_2_, the mean HI titers decreased, despite detectable virus shedding in these groups as well. The ability of the immune system to respond to antigens gradually improves as the MDA level declines in chicks. Therefore, in addition to the antibody level before the challenge, this ability may also have a certain impact on immune responses to the challenge. After the challenge, the drop in HI titers early on (in groups with high titers) could be due to both the blockage of surface epitopes by MDA, thus obstructing the recognition of antigens and reducing the replication of the virus to produce further immune components, and the inability of the immune system to mount an efficient response. And vice versa, the increase in HI titers later on (in groups with lower titers) could be due to less interference from MDA and a better immune response. Moreover, the observations of high CV values (> 15%) of HI titers in groups with titers ranging from 8 log_2_ to 10 log_2_ after the challenge indicated significant diversity in immune responses to infection among individuals in these groups.

In previous studies, Yang et al. [32] and Shen et al. [63] evaluated the protective efficacy of inactivated recombinant genotype VII NDV-matched vaccines (aSG10 and A-VII strains, respectively) against the challenge with genotype VII NDV in layer chickens. Comparing the results obtained in this study with those reported by other authors mentioned above, it is evident that antibodies inherited from hens are less effective than those induced by vaccination in terms of survivability and the number of birds shedding viruses after challenge at the same HI titer. Besides the immature status of the immune system in chicks, this may be related to the difference in immunoglobulin (Ig) components of antibodies of different origins. In chickens, three Ig isotypes, IgM, IgY (avian IgG equivalent), and IgA, are produced by plasmablasts and plasma cells as part of the immune response following infection or vaccination [64]. Approximately 30% of the IgY and 1% of the IgM and IgA antibodies present in the plasma of hens will passively transfer to their offspring [54]. Therefore, IgY, but not IgM and IgA, is the predominant Ig isotype in maternally derived antibodies. It is well known that IgA plays an important role in the inactivation of invading pathogens on mucous membranes. Consequently, maternally derived antibodies containing a small amount of IgA may provide less mucosal protection when compared with vaccine-induced antibodies, especially when birds are infected via the mucosal route with virulent NDV, as was performed in this study.

During the brooding period, chicks are typically vaccinated with ND vaccines at least twice, a primary vaccination with live attenuated vaccine followed by a booster vaccination with inactivated vaccine two weeks later. To avoid the interference of maternally derived antibodies, the primary vaccination is generally administered when maternally derived HI antibody titers fall to around 4 log_2_, while the findings of this study indicate that antibody titers below 7 log_2_ do not provide sufficient clinical protection for chicks. Additionally, birds are unable to generate the antibody levels necessary for clinical protection until at least two weeks after vaccination. Therefore, there is an inevitable “immune gap period” of several weeks during which chicks are more prone to developing clinical disease, even without considering virus shedding, after being infected with field viruses. Thus, strict biosafety measures should be implemented to prevent the entry of field viruses into flocks and to eliminate virulent viruses in the environment of flocks during the brooding period.

## 5. Conclusions

From this study, it is concluded that maternally derived HI antibody levels against NDV in chicks depend on the antibody status in breeder hens. The HI titers in newly hatched chicks are about 1.3 log_2_ lower than those in breeder hens, and approximately 40% of HI antibodies are transferred from hens to their newly hatched offspring. Maternally derived HI antibody levels are slightly raised 2 days after hatching; this level is maintained until 6 days of age and declines gradually afterwards. The half-life of those antibodies is estimated to be about 3.2 days. Maternally derived HI antibodies can effectively protect chicks against clinical disease at a titer of 7 log_2_ or higher, but they are unable to prevent virus shedding or infection, even when the titer is as high as 11 log_2_. These findings will greatly assist producers in determining the immune status of chicks and formulating appropriate vaccination schedules against ND.

## Figures and Tables

**Table 1:** Correlation between HI antibody levels against NDV in chicks and hens.

Breeder Hen ^1^		Chick at Hatching ^2^			Transfer Rate of HI Antibodies (%) ^4^
Number	Antibody Titer (log_2_) ^3^	Number of Birds	Mean Antibody Titer (log_2_)	Lower than That of Hen (log_2_)	
4878	7	7	5.7 ± 0.49	1.3	42.86 ± 12.20
4880	7	6	5.7 ± 0.52	1.3	41.67 ± 12.91
4905	8	7	6.4 ± 0.53	1.6	35.71 ± 13.36
4923	8	6	6.5 ± 0.55	1.5	37.50 ± 13.69
6691	8	7	6.9 ± 0.38	1.1	46.43 ± 9.45
4932	9	9	7.9 ± 0.33	1.1	47.22 ± 8.33
4910	11	8	9.5 ± 0.53	1.5	37.50 ± 13.36
4949	11	6	9.7 ± 0.52	1.3	41.67 ± 12.91

^1^ The breeder hens were 22 weeks old. They were vaccinated at 1 and 4 weeks using the combined live attenuated vaccine against ND and infectious bronchitis (VG/GA strain + H120 strain) (intraocularly and intranasally) and at 5 and 15 weeks using the inactivated recombinant NDV vaccine (A-VII strain, genotype VII) (intramuscularly). ^2^ The chicks were hatched from fertile eggs collected consecutively for 10 days from the hen. ^3^ The antibody titer was detected via HI test using 4 HA units of the genotype VII NDV antigen. ^4^ The transfer rate of HI antibodies = the HI titer in chick/the HI titer in hen × 100%. The transfer rates are expressed as means ± standard deviation (SD). The comparison of transfer rates among hens was made using a single-factor ANOVA test.

**Table 2:** Survivability of chicks with different levels of maternally derived HI antibodies following challenge with virulent genotype VII NDV strain JSC0804.

HI Titer Group (log_2_)	Age of Birds (Day) ^1^	Number of Birds Challenged ^2^	Survival Rate (%) ^3^	Total Survival Rate (%)	Number of Birds with Neurologic Signs/Number of Surviving Birds
11	5	6	100 (6/6) ^4^	100 (6/6) ^a^	0/6
10	5	29	100 (29/29)	100 (31/31) ^a^	0/31
	10	2	100 (2/2)		
9	5	36	100 (36/36)	100 (44/44) ^a^	1/44
	10	8	100 (8/8)		
8	5	31	100 (31/31)	98.3 (57/58) ^ab^	0/57
	10	18	94.4 (17/18)		
	15	9	100 (9/9)		
7	5	10	90.0 (9/10)	95.7 (45/47) ^abc^	1/45
	10	15	93.3 (14/15)		
	15	22	100 (22/22)		
6	10	15	80.0 (12/15)	86.7 (26/30) ^c^	0/26
	15	15	93.3 (14/15)		
5	10	5	100 (5/5)	89.5 (17/19) ^bc^	4/17
	15	12	83.3 (10/12)		
	20	2	100 (2/2)		
4	15	16	50.0 (8/16)	50.0 (10/20) ^d^	3/10
	20	4	50.0 (2/4)		
≤3	20	33	0 (0/33)	0 (0/33) ^e^	- ^5^

^a–e^ Values within a column not sharing a common superscript differ significantly (*p* < 0.05). The differences in survival rates within and among groups were compared using Pearson’s chi-square test. ^1^ The chicks were hatched from the breeder 25-week-old hens. The hens were vaccinated at 1 and 4 weeks using the combined live attenuated vaccine against ND and infectious bronchitis (VG/GA strain + H120 strain) (intraocularly and intranasally) and at 5 and 15 weeks using the inactivated recombinant NDV vaccine (A-VII strain, genotype VII) (intramuscularly). ^2^ Each chick was challenged with 10^6^ ELD_50_ JSC0804 strain intraocularly and intranasally. ^3^ The survival status was observed for 21 days. ^4^ Data in brackets indicate the number of birds survived/the number of birds challenged. ^5^ No surviving birds.

## Data Availability

Not applicable.
